# Machine learning algorithm as a prognostic tool for Epstein-Barr virus reactivation after haploidentical hematopoietic stem cell transplantation

**DOI:** 10.1097/BS9.0000000000000143

**Published:** 2022-12-07

**Authors:** Shuang Fan, Hao-Yang Hong, Xin-Yu Dong, Lan-Ping Xu, Xiao-Hui Zhang, Yu Wang, Chen-Hua Yan, Huan Chen, Yu-Hong Chen, Wei Han, Feng-Rong Wang, Jing-Zhi Wang, Kai-Yan Liu, Meng-Zhu Shen, Xiao-Jun Huang, Shen-Da Hong, Xiao-Dong Mo

**Affiliations:** a Peking University People’s Hospital, Peking University Institute of Hematology, National Clinical Research Center for Hematologic Disease, Beijing Key Laboratory of Hematopoietic Stem Cell Transplantation, Beijing, China; b The Chinese University of Hong Kong, Shenzhen, Shenzhen, China; c National Institute of Health Data Science at Peking University, Peking University Health Science Center, Beijing, China; d Peking-Tsinghua Center for Life Sciences, Academy for Advanced Interdisciplinary Studies, Peking University, Beijing, China; e Research Unit of Key Technique for Diagnosis and Treatments of Hematologic Malignancies, Chinese Academy of Medical Sciences, Beijing, China

**Keywords:** Anti-, thymocyte globulin, Epstein-, Barr virus, Haplo-, identical hematopoietic stem cell transplant, Machine learning, Predictive model

## Abstract

Epstein-Barr virus (EBV) reactivation is one of the most important infections after hematopoietic stem cell transplantation (HSCT) using haplo-identical related donors (HID). We aimed to establish a comprehensive model with machine learning, which could predict EBV reactivation after HID HSCT with anti-thymocyte globulin (ATG) for graft-versus-host disease (GVHD) prophylaxis. We enrolled 470 consecutive acute leukemia patients, 60% of them (n = 282) randomly selected as a training cohort, the remaining 40% (n = 188) as a validation cohort. The equation was as follows: Probability (EBV reactivation) = $\;\frac{1}{1\;\;\;+\;\;\;exp\left(-Y\right)}$, where Y = 0.0250 × (age) – 0.3614 × (gender) + 0.0668 × (underlying disease) – 0.6297 × (disease status before HSCT) – 0.0726 × (disease risk index) – 0.0118 × (hematopoietic cell transplantation-specific comorbidity index [HCT-CI] score) + 1.2037 × (human leukocyte antigen disparity) + 0.5347 × (EBV serostatus) + 0.1605 × (conditioning regimen) – 0.2270 × (donor/recipient gender matched) + 0.2304 × (donor/recipient relation) – 0.0170 × (mononuclear cell counts in graft) + 0.0395 × (CD34+ cell count in graft) – 2.4510. The threshold of probability was 0.4623, which separated patients into low- and high-risk groups. The 1-year cumulative incidence of EBV reactivation in the low- and high-risk groups was 11.0% versus 24.5% (*P* < .001), 10.7% versus 19.3% (*P* = .046), and 11.4% versus 31.6% (*P* = .001), respectively, in total, training and validation cohorts. The model could also predict relapse and survival after HID HSCT. We established a comprehensive model that could predict EBV reactivation in HID HSCT recipients using ATG for GVHD prophylaxis.

## 1. INTRODUCTION

Allogeneic hematopoietic stem cell transplantation (allo-HSCT) significantly improves the survival of patients with acute leukemia (AL).^1^ Recently, with the progression of the transplant technique, human leukocyte antigen (HLA) haplo-identical donors (HIDs) are quantitatively the most important, accounting for 60% of allo-HSCT in China.^2^

Although patients receiving HID HSCT can achieve long-term survival,^3,4^ infection is still the most important cause of transplant-related mortality.^5^ Epstein-Barr virus (EBV) reactivation is one of the most common of these infections. It has been found to be the most important risk factor for EBV-related post-transplant lymphoproliferative disorders (PTLD)^6–8^ and can increase the risk of mortality.^9,10^ Considering that the seroprevalence of EBV in the Chinese population is as high as 90% in children 8 years old or more,^11^ it is important to predict the EBV reactivation after HID HSCT.

Several variables could increase the risk of EBV reactivation after allo-HSCT. Anti-thymocyte globulin (ATG) is the most critical risk factor,^6,12,13^ and approximately 60%–70% of patients receiving ATG during conditioning would experience EBV reactivation after allo-HSCT.^12,14^ In addition, HLA mismatch is another important risk factor for post-transplant EBV reactivation.^6,13^ Thus, patients receiving HID HSCT with an ATG-based regimen would have a high risk of EBV reactivation; however, no accepted risk factors for EBV reactivation have been reported in this population, and there is no comprehensive model to predict EBV reactivation after HID HSCT.

The objective of this article is to establish a comprehensive model, with machine learning, which could predict EBV reactivation after HID HSCT with ATG to counter graft-versus-host disease (GVHD).

## 2. MATERIALS AND METHODS

### 2.1. Study design

This study was conducted on the basis of the transplant database of Peking University, Institute of Hematology; it consisted of 470 consecutive AL patients receiving HID HSCT between January 21, 2020, and May 31, 2021. The information on acute GVHD (aGVHD) has been reported in detail,^15^ and in the present study, the survivors were further followed up to March 1, 2022. The study was conducted in accordance with the Declaration of Helsinki.

### 2.2. Transplant regimens

All patients were treated according to the registered protocol, NCT03756675. The major regimen included cytarabine, busulfan, cyclophosphamide, and semustine for conditioning,^3,16^ using granulocyte colony-stimulating factor-primed peripheral blood (PB) harvests as grafts.^17^ The protocol for GVHD prophylaxis included ATG, cyclosporine A, mycophenolate mofetil, and short-term methotrexate (SDC, Methods, http://links.lww.com/BS/A53).^18–25^

### 2.3. Protocols for EBV monitoring and prevention

Plasma EBV copies were monitored at least weekly until day +100 with quantitative polymerase chain reaction (Q-PCR) analysis. For patients who received systemic immunosuppressive treatments, EBV monitoring was conducted regularly after day +100. If symptoms of suspected virus infection were present, additional detection was performed. The EBV reactivation was defined as more than 1 × 10^3^ copies/mL EBV-DNA in plasma by Q-PCR in 1 test.^26^ The protocols for infection prophylaxis other than EBV and the pre-emptive intervention for EBV reactivation are shown in the SDC, Methods (http://links.lww.com/BS/A53).

### 2.4. Building machine learning models

**Figure 1.**
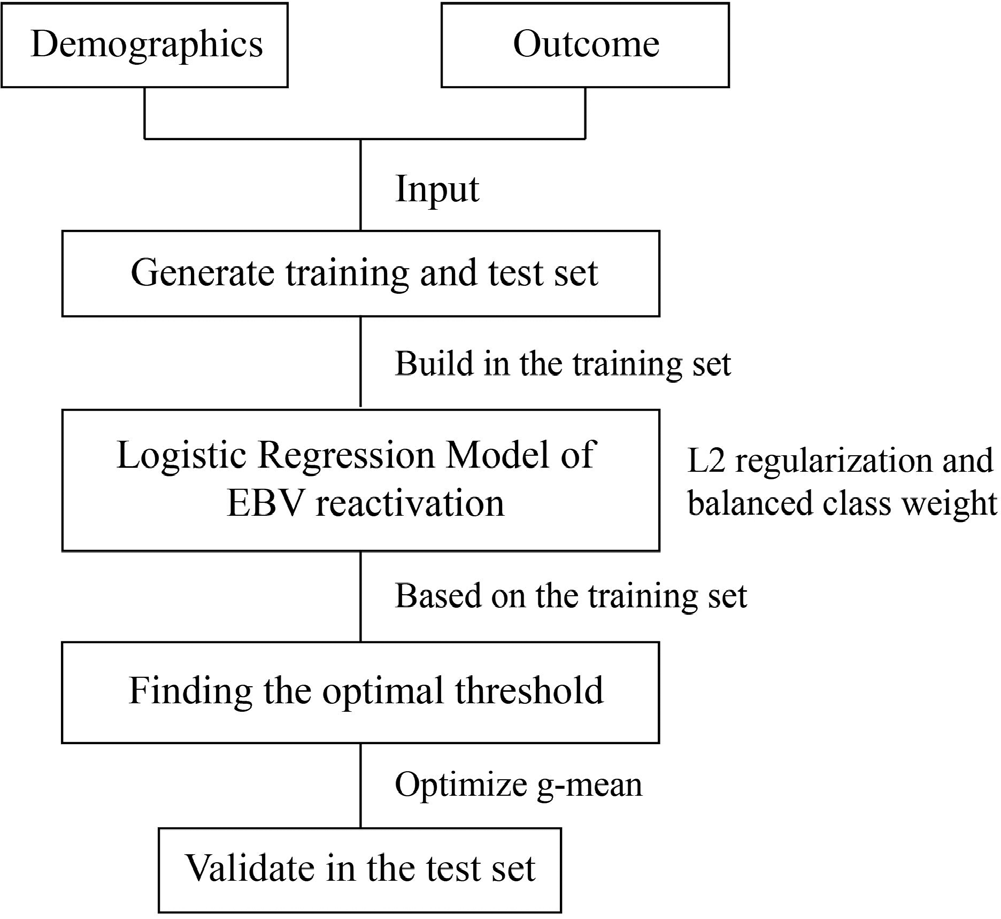
Flow diagram of building the machine learning model. EBV = Epstein-Barr virus.

Our method consisted of 2 steps: building the logistic regression model and ascertaining the optimal threshold (Fig. 1; SDC, Methods, http://links.lww.com/BS/A53; and SDC, Table S1, http://links.lww.com/BS/A53).^27–31^

Of the entire study population, 60% were randomly selected (ie, n = 282) as the training cohort; the remaining 40% were used as the validation cohort (n = 188). For the primary outcome (EBV reactivation), we performed the model-building steps in the training cohort and verified the model in the validation cohort. We also identified the sensitivity, specificity, area under the curve (AUC) score and accuracy score in both data cohorts.

#### 2.4.1. Building models

We utilized logistic regression models with L2 regularization for the prediction. The model is illustrated in equation (1):


$$\begin{array}{l} P\left(Y=1\;|\;X=x\right)=\frac{1}{1+exp\left(-(w^{T}x+c)\right)}\cdots \left(1\right) \end{array}$$

In equation (1), w is the coefficient to be trained, which requires the following objective function to be minimized:


$$\begin{array}{l} \underset{w,c}{\min}\;\frac{1}{2}w^{T}w+C\overset{n}{\underset{i=1}{\sum }}\log(\exp\left(-y_{i}\left(X_{i}^{T}w+c\right)+1\right)\cdots \left(2\right) \end{array}$$

During the optimization procedure, an inappropriate imbalance between the sizes of the positive and negative samples was found. We adjusted weights to each sample when conducting optimization as in equation (3):


$$\begin{array}{l} n\_samples/\left(n\_classes\times \;\left[n\_negative,\;n\_positive\right]\right)\cdots \left(3\right) \end{array}$$

We utilized sklearn v1.0.2 with Python 3.9 to build the models based on the anaconda3 development platform. The model parameters “class_weight” and “max_iter” are set to be “balanced” and 1000, respectively.^32,33^

#### 2.4.2. Finding the optimal threshold

According to equation (1), the output of the logistic regression model should be between 0 and 1. To further specify the prediction results, determining the threshold for outputting negative or positive became significant. In this article, we drew receiver operating characteristic (ROC) curves^30^ and calculated the g-mean for each threshold.^31^ We chose the one with the largest g-mean to be the optimal threshold.

#### 2.4.3. Evaluation for model

ROC-AUC was defined as the AUC of the tpr/fpr at thresholds ranging from 0 to 1. The confusion matrix was a 2 × 2 table for summarizing the prediction results. In addition, we normalized the count values by the number of True Label (Outcome) or the number of Predicted Label (Prediction).

The detailed information for the setting of equations is shown in the SDC, Methods (http://links.lww.com/BS/A53).

### 2.5. Definitions

The definitions for hematopoietic cell transplantation-specific comorbidity index (HCT-CI) and disease risk index (DRI) were as in previous studies.^34,35^ The definitions for engraftment, nonrelapse mortality (NRM), relapse, overall survival (OS), and leukemia-free survival (LFS) are shown in the SDC, Methods (http://links.lww.com/BS/A53).

### 2.6. Statistical methods

The primary outcome was EBV reactivation. The secondary outcomes included relapse, NRM, OS, and LFS.

We used the Mann–Whitney *U* test to compare continuous variables and the χ^2^ and Fisher exact tests for categorical variables. The Kaplan–Meier method was used to estimate the probability of OS and LFS. We used competing risk analyses to calculate the cumulative incidence of EBV reactivation, NRM, and relapse.^36^ Testing was 2-sided at the *P* < .05 level. Statistical analysis was performed on R software (version 4.2.0) (http://www.r-project.org) and SPSS 26.0 software (SPSS, Chicago, Illinois).

## 3. RESULTS

### 3.1. Characteristics of patients

Table 1 shows the characteristics of the training and validation cohorts. The detailed information of engraftment and aGVHD have been previously reported by Shen et al.^15^ A total of 438 patients (92.9%) survived until the last follow-up. The median duration of follow-up was 483 days (range, 39–770 d). The probabilities of NRM, relapse, OS and LFS at 1 year after HID HSCT were 3.9% (95% CI, 2.1%–5.7%), 8.7% (95% CI, 6.1%–11.2%), 93.9% (95% CI, 91.8%–96.1%), and 87.4% (95% CI, 84.4%–90.5%), respectively.

**Table 1:** Patient characteristics.

Characteristics	Training cohort (n = 282)	Validation cohort (n = 188)	*P*
Median age at allo-HSCT, y (range)	27.5 (1–65)	30.0 (1–66)	.514
Gender, n (%)			.618
Male	166 (58.9)	115 (61.2)	
Female	116 (41.1)	73 (38.8)	
Underlying disease, n (%)			.495
Acute myeloid leukemia	162 (57.4)	102 (54.3)	
Acute lymphoblastic leukemia	120 (42.6)	86 (45.7)	
Disease status before allo-HSCT, n (%)			.922
CR1	271 (96.1)	181 (96.3)	
>CR1	11 (3.9)	7 (3.7)	
Disease risk index before allo-HSCT, n (%)			.395
Low risk	14 (5.0)	10 (5.3)	
Intermediate risk	209 (74.1)	145 (77.1)	
High risk	59 (20.9)	33 (17.6)	
HCT-CI scores before allo-HSCT, n (%)			.514
0 (low risk)	204 (72.3)	138 (73.4)	
1–2 (intermediate risk)	54 (19.1)	43 (22.9)	
≥3 (high risk)	24 (8.5)	7 (3.7)	
Number of HLA-A, HLA-B, HLA-DR mismatches, n (%)			.575
1 locus	8 (2.8)	3 (1.6)	
≥2 loci	274 (97.2)	185 (98.4)	
EBV serostatus before HSCT, n (%)			.730
Donor+/recipient–	9 (3.2)	4 (2.1)	
Donor+/recipient+	259 (91.8)	173 (92.0)	
Donor–/recipient+	14 (5.0)	11 (5.8)	
Conditioning regimen, n (%)			.049
Chemotherapy-based regimen	271 (96.1)	187 (99.5)	
TBI-based regimen	11 (3.9)	1 (0.5)	
Donor/recipient gender matched, n (%)			.408
Female donor/male recipient combination	55 (19.5)	31 (16.5)	
Others	227 (80.5)	157 (83.5)	
Donor/recipient relation, n (%)			.031
Maternal donor	30 (10.6)	8 (4.3)	
Collateral donor	8 (2.8)	4 (2.1)	
Others	244 (86.5)	176 (93.6)	
MNC counts in graft, median (range, ×10^8^/kg)	9.35 (4.15–27.52)	9.03 (5.76–15.94)	.335
CD34+ cell counts in graft, median (range, ×10^6^/kg)	3.79 (0.67–29.35)	3.84 (1.15–15.91)	.595
Median follow-up of survivors, d (range)	468.5 (39–770)	497 (66–768)	.437

allo-HSCT = allogeneic hematopoietic stem cell transplantation, CR = complete remission, EBV = Epstein-Barr virus, HCT-CI = hematopoietic cell transplantation-specific comorbidity index, HLA = human leukocyte antigen, MNC = mononuclear cell, TBI = total body irradiation.

### 3.2. EBV characteristics

A total of 80 patients (17.0%) showed EBV reactivation. The median time from HSCT to EBV reactivation was 52 days (range, 20–579 d). The initial and highest plasma levels of EBV-DNAemia were 1.62 × 10^3^ copies/mL(range, 1.00–25.10 × 10^3^ copies/mL) and 2.96 × 10^3^ copies/mL (range, 1.02–56.00 × 10^3^ copies/mL), respectively. The cumulative incidence of EBV reactivation at 1 year after HID HSCT was 16.6% (95% CI, 13.3%–20.0%). The number of patients showing PTLD after EBV reactivation was 12.

### 3.3. Predictive model for EBV reactivation

Our equation was as follows:

Probability (EBV reactivation) = $\;\frac{1}{1\;\;\;+\;\;\;exp\left(-Y\right)}$

where

Y = 0.0250 × (patient age) – 0.3614 × (patient gender) + 0.0668 × (underlying disease) – 0.6297 × (disease status before HSCT) – 0.0726 × (DRI) – 0.0118 × (HCT-CI score) + 1.2037 × (HLA disparity) + 0.5347 × (EBV serostatus) + 0.1605 × (conditioning regimen) – 0.2270 × (donor/recipient gender matched) + 0.2304 × (donor/recipient relation) – 0.0170 × (mononuclear cell count in graft) + 0.0395 × (CD34+ cell count in graft) – 2.4510 (Table 2). The threshold of probability was set as 0.4623, and the g-mean was 0.648; thus, the patients could be separated into low- and high-risk groups by the threshold values.

**Table 2:** Variables for building machine learning models.

Variables	Assignment for variables
Age (y)	Numerical value
Gender	Male = 0; female = 1
Underlying disease	Acute myeloid leukemia = 0; acute lymphoblastic leukemia = 1
Disease status before HSCT	CR1 = 0; >CR1 = 1
DRI	Low risk = 0; intermediate risk = 1; high risk = 2
HCT-CI score	Numerical value
HLA disparity	1 locus = 0; ≥2 loci = 1
EBV serostatus	D+/R– = 0; D+/R+ = 1; D–/R+ = 2
Conditioning regimen	TBI-based = 0; chemotherapy-based = 1
Donor/recipient gender matched	Others = 0; female donor/male recipient = 1
Donor/recipient relation	Immediate related donors, others = 0; immediate related donors, maternal donors = 1; collateral related donors = 2
Mononuclear cell counts in graft (×10^8^/kg)	Numerical value
CD34^+^ cell counts in graft (×10^6^/kg)	Numerical value

CR = complete remission, D = donor, DRI = disease risk index, EBV = Epstein-Barr virus, HCT-CI = hematopoietic cell transplantation-specific comorbidity index, HLA = human leukocyte antigen, HSCT = hematopoietic stem cell transplant, R = recipient, TBI = total body irradiation.

**Figure 2.**
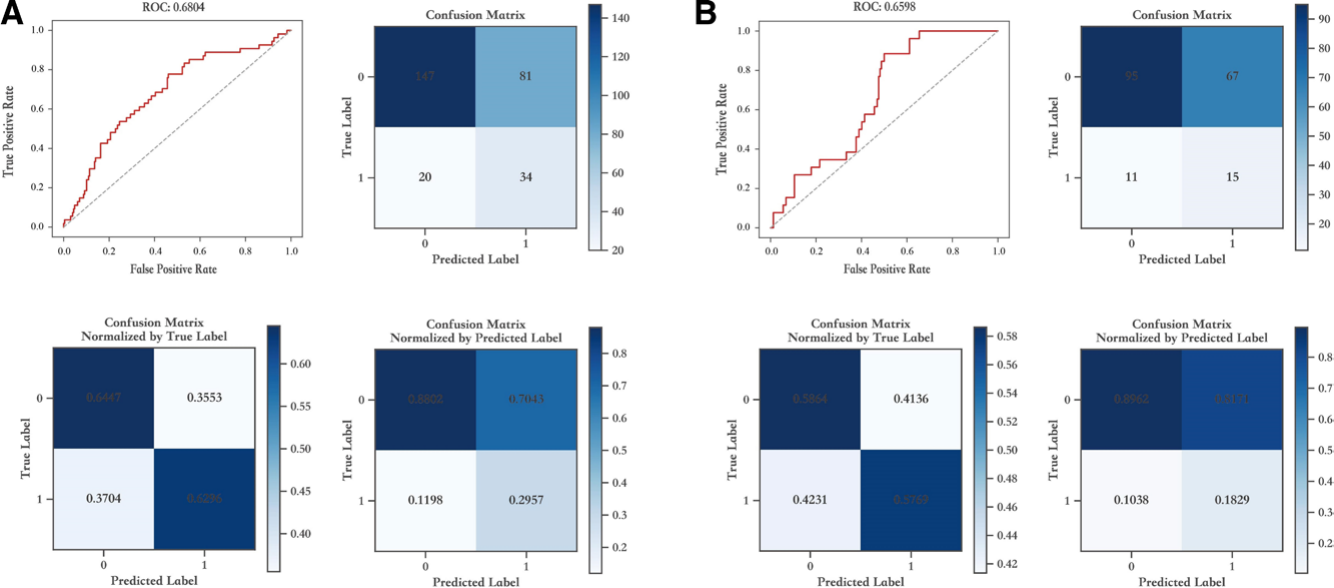
**ROC curve and confusion matrix for EBV reactivation model. (A) in the training and (B) in the validation cohort.** EBV, Epstein-Barr virus..

In the training cohort, the sensitivity, specificity, AUC score and accuracy score were 0.7593, 0.5395, 0.6804, and 0.5816, respectively (Fig. 2A and SDC, Table S2, http://links.lww.com/BS/A53). In the validation cohort, the sensitivity, specificity, AUC score, and accuracy score were 0.8846, 0.4938, 0.6598, and 0.5479, respectively (Fig. 2B and SDC, Table S3, http://links.lww.com/BS/A53).

### 3.4. Predicted value of our comprehensive model in the total cohort

**Figure 3.**
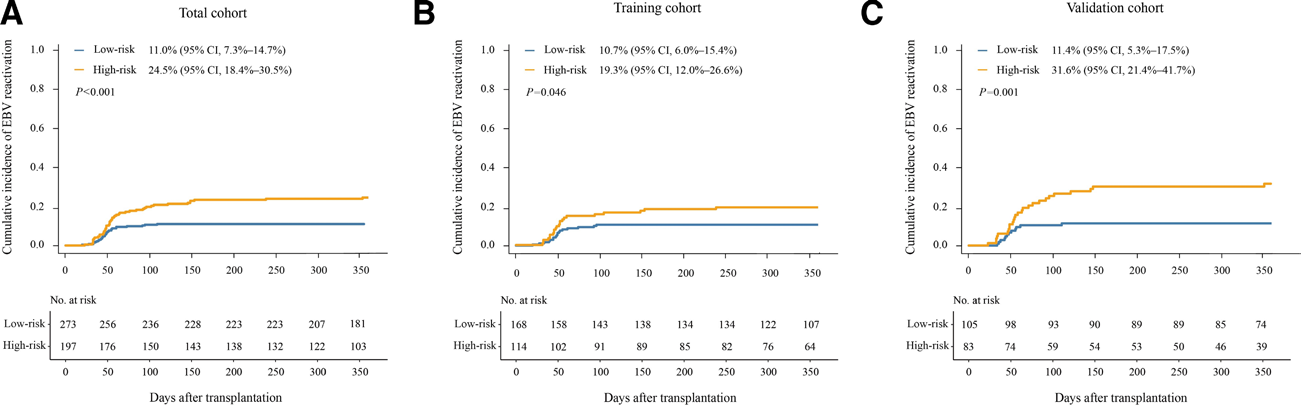
**The 1-year cumulative incidence of EBV reactivation in the low- and high-risk groups. (A) in total cohorts, (B) in training cohorts, and (C) in validation cohorts.** EBV, Epstein-Barr virus..

The 1-year cumulative incidence of EBV reactivation after HID HSCT was 24.5% (95% CI, 18.4%–30.5%) and 11.0% (95% CI, 7.3%–14.7%), respectively, in the high- and low-risk groups (*P* < .001; Fig. 3A), in the total cohort.

In the training cohort, the cumulative incidence of EBV reactivation at 1 year after HID HSCT was 19.3% (95% CI, 12.0%–26.6%) and 10.7% (95% CI, 6.0%–15.4%), respectively, in the high- and low-risk groups (*P* = .046; Fig. 3B). In the validation cohort, the cumulative incidence of EBV reactivation at 1 year after HID HSCT was 31.6% (95% CI, 21.4%–41.7%) and 11.4% (95% CI, 5.3%–17.5%), respectively, in the high- and low-risk groups (*P* = .001; Fig. 3C).

The cumulative incidence of EBV reactivation at 1 year after HID HSCT was significantly higher in the high-risk group than in the low-risk group in the patients with HCT-CI scores of 0 (SDC, Figure S1, http://links.lww.com/BS/A53). The low-risk group showed a trend to lower incidence of EBV reactivation compared with the high-risk group for patients with HCT-CI scores of ≥1 (SDC, Figure S2, http://links.lww.com/BS/A53).

The cumulative incidence of EBV reactivation with clinical meaning at 1 year after HID HSCT was 4.4% (95% CI, 2.0%–6.8%) and 9.1% (95% CI, 5.1%–13.2%) (*P* = .039), respectively, in the low- and high-risk groups.

The cumulative incidence of PTLD at 1 year after HID HSCT was 1.5% (95% CI, 0.0%–2.9%) and 4.1% (95% CI, 1.3%–6.8%) (*P* = .078), respectively, in the low- and high-risk groups.

### 3.5. Predicted value of our comprehensive model in patients without or with cytomegalovirus

**Figure 4.**
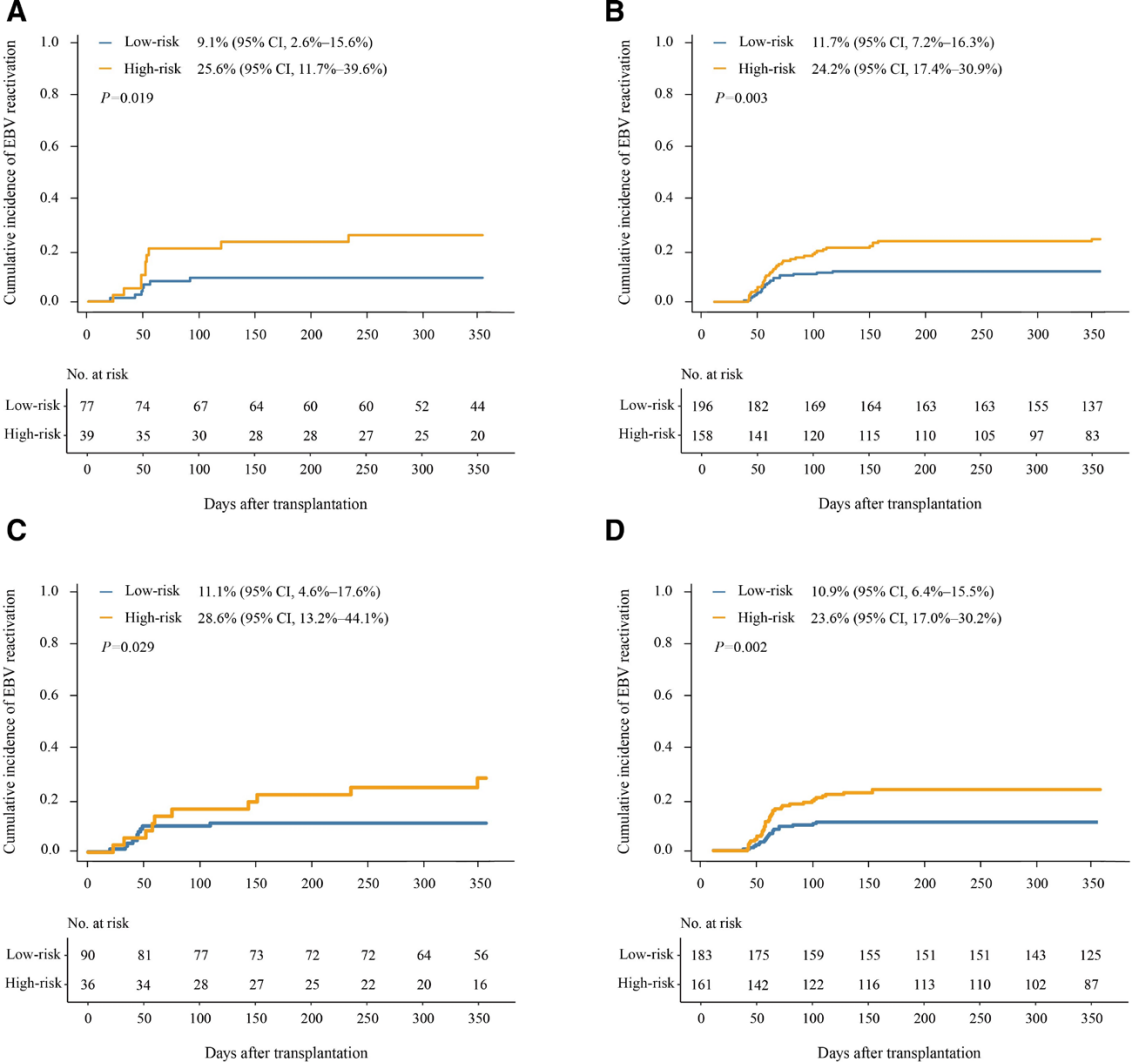
**The 1-year cumulative incidence of EBV reactivation. (A) in patients without CMV, (B) in patients with CMV, (C) in patients with grade II to IV aGVHD, and (D) in patients without aGVHD or with grade I aGVHD.** EBV, Epstein-Barr virus; aGVHD, acute graft-versus-host disease.

The cumulative incidence of EBV reactivation at 1 year after HID HSCT was 9.1% (95% CI, 2.6%–15.6%) and 25.6% (95% CI, 11.7%–39.6%), respectively, in the low- and high-risk groups (*P* = .019) in those without cytomegalovirus (CMV)-DNAemia (n = 116) (Fig. 4A).

In patients with CMV-DNAemia (n = 354), the cumulative incidence of EBV reactivation at 1 year after HID HSCT was 11.7% (95% CI, 7.2%–16.3%) and 24.2% (95% CI, 17.4%–30.9%), respectively, in the low- and high-risk groups (*P* = .003; Fig. 4B).

### 3.6. Predicted value of our comprehensive model in patients without or with severe aGVHD

In patients with grade II to IV aGVHD (n = 126), the cumulative incidence of EBV reactivation at 1 year after HID HSCT was 28.6% (95% CI, 13.2%–44.1%) and 11.1% (95% CI, 4.6%–17.6%), respectively, in the high- and low-risk groups (*P* = .029; Fig. 4C).

In patients without aGVHD or with grade I aGVHD (n = 344), the cumulative incidence of EBV reactivation at 1 year after HID HSCT was 23.6% (95% CI, 17.0%–30.2%) and 10.9% (95% CI, 6.4%–15.5%) (*P* = .002), respectively, in the high- and low-risk groups (Fig. 4D).

### 3.7. Secondary outcomes after HID HSCT

**Figure 5.**
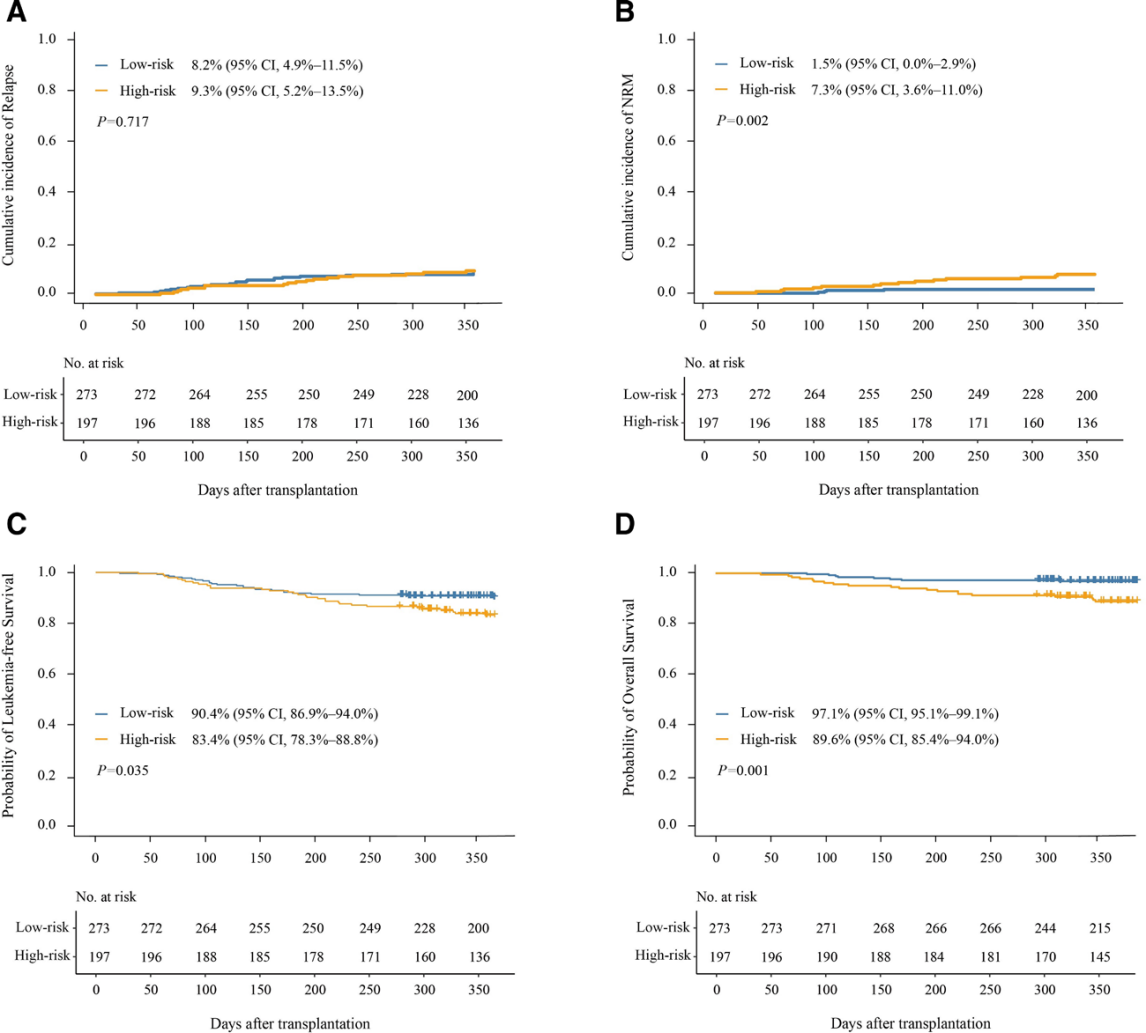
**The 1-year cumulative incidence of secondary outcomes after HID HSCT in the low- and high-risk groups. (A) relapse, (B) NRM, (C) LFS, and (D) OS.** NRM, non-relapse mortality; LFS, leukemia-free survival; OS, overall survival.

The cumulative incidence of NRM, OS and LFS at 1 year after HID HSCT for patients in the high-risk group was significantly poorer than in low-risk group. The incidence of relapse was comparable between the groups (Fig. 5).

## 4. DISCUSSION

In the present study, we propose a predictive model for EBV reactivation after HID HSCT with the help of machine learning. It can categorize the patients into low- and high-risk groups for EBV reactivation. We first integrated different variables and established a comprehensive model that could effectively predict EBV reactivation in HID HSCT recipients with ATG for GVHD prophylaxis.

Several studies have already identified the risk factors for EBV reactivation after allo-HSCT.^9,10,12,13,26^ However, only male patients and intensified conditioning regimens were potential risk factors besides ATG and HLA mismatched donors, and using 1 or 2 variables to predict EBV reactivations was distinctly insufficient for HID HSCT recipients on ATG-based regimens. According to machine learning theory, adding more variables can increase the capacity and performance of the upper boundary of the predictive model.^37,38^ Thus, our comprehensive model included 13 demography, disease, and transplant characteristics. However, the large number of variables may induce overfitting in the training set.^39^ Our strategy is to add an L2 regularization term as shown in the objective function (equation 2). By introducing a regularization term to the objective function, the weights for coefficients become more balanced, thereby reducing the risk of overfitting.^40^ In addition, an imbalance problem was found between the sizes of the positive and negative samples. We adopted adjusted weights (equation 3) during the optimization procedure.^41^ In this way, we enhanced the weights for the positive samples to alleviate these adverse effects. Both methods contributed to a more generalizable and robust model. Our strategy was therefore in a step-wise manner and ensured the stability of the feature-selection process. In addition, we added a penalty function of the regularization term in the model optimization process, which can decrease the risk of overfitting the training data.

Considering that not all the patients would experience particular post-transplant complications (eg, aGVHD), we only enrolled the common transplant characteristics, and the model could be used in patients without post-transplant complications. For example, we observed that this model could predict EBV reactivation in patients without CMV-DNAemia or without severe aGVHD. This may help to increase the generality of our model.

In the present study, we observed that high-risk patients showed a higher incidence of NRM and a lower probability of survival compared with low-risk patients. Some studies also reported that EBV reactivation could increase the risk of mortality after allo-HSCT.^6,9,10^ This also supported the clinical significance of our predicted model.

Several studies reported that prophylactic rituximab treatment^42^ or EBV-specific T-cell infusions^43^ could decrease the risk of EBV reactivation. Considering that the median time from HSCT to EBV reactivation was nearly 2 months, we may have plenty of chances to conduct risk stratification-directed EBV prophylaxis after HID HSCT in high-risk patients on the basis of our predicted model, while the low-risk patients can avoid unnecessary treatment-related toxicities.

Regarding the limitations of our study, although we confirmed the model in the validation cohort successfully, this cohort was relatively small. Also, it did not enroll patients receiving unrelated-donor or identical-sibling-donor allo-HSCT with ATG for prophylaxis. Our cohort did not enroll HID HSCT with post-transplant cyclophosphamide either, although EBV reactivation is relatively rare in these patients.^44^ Thus, the model should be further evaluated by independent cohorts in multicenter studies with other donor types and transplant regimens. In the present study, only 1 patient died of PTLD. It is still premature for our model to predict EBV-related deaths, and this should be investigated further. Finally, only 3 patients showed new-onset EBV reactivation after chronic GVHD (cGVHD). We could not further identify the efficacy of our model in patients with cGVHD, and this requires additional study.

## 5. CONCLUSIONS

We have established a comprehensive model that could predict EBV reactivation in HID HSCT recipients using ATG for GVHD prophylaxis with machine learning. This is the first predictive model for these patients, who have a high risk of EBV reactivation, and it can be popularized easily. In future, prospective, multicenter studies can further confirm the efficacy of our predictive model. It can also help to conduct risk stratification-directed EBV prophylaxis after HID HSCT.

## ACKNOWLEDGMENTS

This work was supported by the National key research and development plan of China (2022YFC2502606), the Program of the National Natural Science Foundation of China (grant number 82170208), the Foundation for Innovative Research Groups of the National Natural Science Foundation of China (grant number 81621001), the CAMS Innovation Fund for Medical Sciences (CIFMS) (grant number 2019-I2M-5-034), the Key Program of the National Natural Science Foundation of China (grant number 81930004), and the Fundamental Research Funds for the Central Universities, National Natural Science Foundation of China (No. 62102008).
